# Mechanisms and Role of Nitric Oxide in Phytotoxicity-Mitigation of Copper

**DOI:** 10.3389/fpls.2020.00675

**Published:** 2020-05-29

**Authors:** Bilal A. Rather, Asim Masood, Zebus Sehar, Arif Majid, Naser A. Anjum, Nafees A. Khan

**Affiliations:** Plant Physiology and Biochemistry Laboratory, Department of Botany, Aligarh Muslim University, Aligarh, India

**Keywords:** copper, nitric oxide, oxidative stress, photosynthesis, Cu-stress mitigation

## Abstract

Phytotoxicity of metals significantly contributes to the major loss in agricultural productivity. Among all the metals, copper (Cu) is one of essential metals, where it exhibits toxicity only at its supra-optimal level. Elevated Cu levels affect plants developmental processes from initiation of seed germination to the senescence, photosynthetic functions, growth and productivity. The use of plant growth regulators/phytohormones and other signaling molecules is one of major approaches for reversing Cu-toxicity in plants. Nitric oxide (NO) is a versatile and bioactive gaseous signaling molecule, involved in major physiological and molecular processes in plants. NO modulates responses of plants grown under optimal conditions or to multiple stress factors including elevated Cu levels. The available literature in this context is centered mainly on the role of NO in combating Cu stress with partial discussion on underlying mechanisms. Considering the recent reports, this paper: (a) overviews Cu uptake and transport; (b) highlights the major aspects of Cu-toxicity on germination, photosynthesis, growth, phenotypic changes and nutrient-use-efficiency; (c) updates on NO as a major signaling molecule; and (d) critically appraises the Cu-significance and mechanisms underlying NO-mediated alleviation of Cu-phytotoxicity. The outcome of the discussion may provide important clues for future research on NO-mediated mitigation of Cu-phytotoxicity.

## Introduction

The increasing industrialization, hasty urbanization together with excessive use of chemical fertilizers and sewage sludge/water led to the severe contamination of soils with varied metals and metalloids (Nagajyoti et al., 2010; Brunetto et al., 2016; Asgher et al., 2018a, b). Among metals, copper (Cu) is an essential metal for plants, and promotes therein growth and development at 9.0 mg kg^–1^ (Havlin et al., 1999). The average content of Cu in plant tissues is 10 μg g^–1^ dry weight; whereas, the precarious Cu concentration in nutrient media ranges between 10^–14^ and 10^–16^ M at which its deficiency has been noted (Baker and Senef, 1995). Cu is involved in the photosynthetic electron transport and redox reactions and it also acts as a cofactor in Cu/Zn–superoxide dismutase (Cu/Zn-SOD) (Bowler et al., 1994; Ouzounidou et al., 1995; Raven et al., 1999; Adrees et al., 2015). However, elevated Cu concentration can induce oxidative stress mainly through increased generation of reactive oxygen species (ROS) and thereby inhibit plant growth and productivity (Piotrowska-Niczyporuk et al., 2012; Thounaojam et al., 2012; Adrees et al., 2015; Chen et al., 2015).

Plants adopt different strategies to overcome elevated Cu concentration-caused toxicity. The list of these strategies includes the increased nutrient assimilation, induction in the antioxidant defense system, and the activation of biochemical and physiological processes such as increased signaling through associated plant growth regulators (PGRs). PGRs such as auxins (AUXs), cytokinins (CKs), gibberellins (GAs), brassinosteroids (BRs), ethylene (ET), jasmonic acids (JA), polyamines (PA), salicylic acid (SA), nitric oxide (NO), and strigolactones modulate physiological/biochemical and genetic processes and improve plant tolerance to major abiotic stresses including metals/metalloids (Maksymiec et al., 2007; Meng et al., 2009; Khan et al., 2012, 2015, 2016; Masood et al., 2012, 2016; Per et al., 2016, 2017a; Asgher et al., 2018a, b).

Earlier known as a vital signaling and effector molecule in animals, NO is known to occur also in plants, and act therein as a short-lived multifunctional gaseous signaling molecule (Astier et al., 2017). NO controls overall plant growth and the developmental processes starting from germination to senescence (Hu et al., 2007; Corpas and Barroso, 2015b). In particular, recent studies have shown that both production and signaling of NO are involved in the stress-acclimation processes in plants (Khan et al., 2012; Asgher et al., 2017; Kushwaha et al., 2019; Santisree et al., 2019). However, literature available in context with NO and plants is centered mainly on NO synthesis and its multitasking signaling in plants (Domingos et al., 2015; Astier et al., 2017). Additionally, literature in this context also reflected the role of exogenously applied NO in combating Cu stress with partial discussion on underlying potential mechanisms (Khan et al., 2012).

Given above, this paper is aimed to: (i) overview the aspects of the uptake, transport, and role of Cu in photosynthesis and nutrient-use-efficiency; (ii) highlight major aspects of Cu-phytotoxicity; (iii) discuss NO as a signaling molecule; and (iv) critically appraise the Cu-significance and mechanisms underlying NO-mediated alleviation Cu-toxicity in plants. Important clues for future research in this direction with the outcome of the facts are also discussed herein.

## Copper in Higher Plants

Copper (Cu) is an essential trace element of most living organisms on the earth including plants, where >30 types of proteins are known to possess Cu as their structural constituent (Cohu and Pilon, 2010; Anjum et al., 2015b). As an essential micronutrient for plants, a minimum amount of Cu ensures different cellular functions. However, an excess uptake of Cu in plants may cause detrimental effects in metabolic functions and even risk to their survival (Adrees et al., 2015; Ambrosini et al., 2018; Marastoni et al., 2019b). In plants, Cu exists in two common oxidation states namely, Cu^2+^ and Cu^+^ ions. Cu^2+^ frequently prefers coordination with oxygen in aspartic and glutamic acid and with nitrogen in histidine side chains. On the other, Cu^+^ has a higher affinity with the sulfur in methionine or cysteine. The list of major Cu-containing proteins in plants includes plastocyanin, cytochrome-*c* oxidase (COX), ethylene receptors, Cu/Zn–superoxide dismutase (Cu/Zn SOD), tyrosinase, plantacyanin, phenol oxidase, laccase, ascorbate, and amine oxidase (Table 1). Cu mainly contributes in the transport of electrons in chloroplasts and mitochondria. Plastocyanin is one of the most abundant Cu proteins in photosynthetic tissues (Weigel et al., 2003). It is located in thylakoid lumen of chloroplasts and is responsible for the transport of electrons from cytochrome b6f complex to P700^+^. Though in some algae this function can be served by a heme-containing cytochrome c6, information is scanty on the ortholog that can mimic the same function of plastocyanin in higher plants (Schubert et al., 2002; Wise and Hoober, 2007). Cu also serves as a critical co-factor in the mitochondria as a respiratory chain enzyme cytochrome *c* oxidase (COX). Composed of 12-14 sub-units, plant COX is the terminal enzymatic complex IV of the mitochondrial respiratory chain (Millar et al., 2004). Another major Cu-binding protein in plants is Cu/Zn-superoxide dismutase (Cu/Zn-SOD) that occurs in cytosol, chloroplasts, and peroxisomes. Arabidopsis possess three isoforms of CuZn-SOD, where CSD1 is located in the cytoplasm; CSD2 in chloroplast stroma, and CSD3 is a peroxisomal isoform having a minor activity (Kliebenstein et al., 1998). Another Cu-protein plantacyanin belongs to phytocyanin family of blue Cu containing proteins. Based on their magnetic and spectroscopic properties, plantacyanins are classified as Type I Cu proteins with size about 10–22 kDa (de Rienzo et al., 2000), and are primarily present in the cell wall. They are believed to act as transporters of electrons between a donor and acceptor. Plantacyanins are expressed in plants exposed to stresses such as high/low temperature, heavy metals, and high salinity, and are involved in plant-tolerance to these stresses (Maunoury and Vaucheret, 2011; Feng et al., 2013). Cu-dependent protein laccase belongs to the large group of multi-copper oxidases (MCOs) and is involved in polymerization (McCaig et al., 2005; Printz et al., 2016). Cu-dependent amine oxidases (CuAO) are among the amine oxidases and are associated with the cell wall, and flavin-containing polyamine oxidases. In plants, CuAO catalyzes the oxidation of putrescine that produces H_2_O_2_ involved in cell wall maturation, lignification, and programmed cell death (Cona et al., 2006). Polyphenol oxidase and ascorbate oxidases (AO) also belong to Cu-containing MCOs. Localized in the apoplast, AOs oxidize ascorbate into water and monodehydroascorbate, and thereby regulate its redox state (Kaur and Nayyar, 2014). AOs also modulate cell division and cell expansion via L-ascorbic acid (L-AA) redox repair (Kerk and Feldman, 1995; Kato and Esaka, 1999). Polyphenol oxidases are found in thylakoids, where these are involved in the defense mechanisms against pests and pathogens (Constabel and Barbehenn, 2008). In addition, Cu also plays an important role in cell signaling as the part of receptor proteins for ethylene sensing (Rodrıguez et al., 1999). Cu-homeostasis is mainly regulated by the transcription factor SQUAMOSA PROMOTER BINDING PROTEIN-LIKE7 (SPL7). Through activating the transcription of plasma membrane COPT transporter genes (*COPT1*, *COPT2*, and *COPT6*), SPL7 modulates the Cu-uptake and homeostasis under Cu-deficiency (Yamasaki et al., 2009; Bernal et al., 2012). Thus, these requirements make Cu an ideal metal for normal functioning, growth and development in plants.

**TABLE 1:** Summary of copper-associated proteins and their functions in plants.^1^

**Type**	**Protein**	**Function**
**Cell Surface/Secretory Compartment Transporters and Receptors**		
	P1B-Type ATPases	Proteins concerned with export of Cu^+^
	Ctr (copper transporter)	Proteins involved in import of Cu^+^
	Ethylene receptor	Cu acts as a cofactor and activates ethylene signaling
**Electron transfer/Blue Cu proteins**		
	Cytochrome *c* oxidase	Plays an important role in the last step of respiration
	Plastocyanin	Electron transfer during photosynthesis
**Free Radical Scavenging**		
	Cu/Zn SOD	Scavenger of free radicals
**Oxidase**		
	Laccase	Oxidative de-amination of polyamines
	Ascorbate oxidase	Regulates redox state of the cell
	Amine oxidase	Involved in cell wall maturation, lignification, Oxidizes diamines
	Polyphenol oxidase	Plays an important role in defensive mechanisms against pests and pathogens
**Transcriptional regulators**		
	Spl7	Transcriptional activator which gets activated in response to reduced Cu levels.
**Chaperons/Storage**		
	Atx1 (Antioxidant protein 1)	A metal chaperone carrying Cu to P-Type ATPases
	Ccs (Cu chaperone for superoxide dismutase)	Transports Cu to Cu/Zn SOD1

*^1^Based on the literature appraised in the paper.*

On the contrary, the condition of both Cu-deficiency and -elevation can bring severe consequences in plants (Yruela, 2005). Plants produced under Cu-deficiency showed alteration in the photosynthetic transport chain and reduction in the non-photochemical quenching, which is mainly due to inhibition in the function of plastocyanin (Abdel-Ghany and Pilon, 2008). On the otherside, Cu in excess causes significant toxicity and even the arrest of cellular metabolism in plants. In particular, photosynthetic electron transport is the main target under both Cu-deficiency and as well as in excess Cu. Therefore, it is essential to ensure adequate Cu-uptake and distribution in order to minimize its deleterious phytotoxic effects that in turn would regulate various homeostatic processes at cellular and whole plant levels.

## Copper Uptake and Transport

Higher plants mainly take Cu in the form of Cu^2+^ ions from the rhizosphere, where the binding of Cu with various ligands facilitates the process (Welch et al., 1993). The studies on Cu uptake and transport into or within the cells are still in infancy. However, the successful implementation of advanced tools helped in uncovering transport process in yeast and other eukaryotic organisms (Nelson, 1999; Nevitt et al., 2012). Maintenance or correct regulation of Cu-homeostasis under Cu-regimes is governed by a complex system of metal-trafficking pathways available in higher plants. Plants possess a number of Cu-transporters (COPT; *COPT1-6*) involved in the uptake of Cu and secretion of metal ions (Puig et al., 2007; Andrés-Bordería et al., 2017; Andrés-Colás et al., 2018).

Current understandings on COPTs came into light as a result of having sequence homology with the eukaryotic Cu-transporters (named Ctr) and functional complementation in yeast (Puig and Thiele, 2002; Puig et al., 2007; Puig, 2014; Andrés-Bordería et al., 2017). All the members of this family contain three predicted transmembrane (TM) segments. The majority of the COPTs exhibit N-terminus methionine and histidine-rich putative metal-binding domains (Puig and Thiele, 2002; Klomp et al., 2003). In *Arabidopsis* genome, there occurs six COPT genes (*COPT 1-6*) encoding COPT transporters. COPT1, one of the most characterized members of the Cu-transporter family has been reported to permit the entry of Cu into the cells from outside to the cytoplasm (Kampfenkel et al., 1995; Sancenón et al., 2003). In addition, owing to its low Michaelis constant (KM) value, COPT1 transporter has also been reported to exhibit its high specificity for Cu^2+^ ion (Eisses and Kaplan, 2002; Sancenón et al., 2003). High specificity toward Cu^2+^ ions has also been reported for COPT2 and COPT 6 transporters (Jung et al., 2012; Garcia-Molina et al., 2013; Perea-García et al., 2013; Aguirre and Pilon, 2016). Potentially involved in the intracellular transport of Cu, COPT3 and COPT5 transporters possess one each of methionine and a histidine-rich box. Methionine residues and motifs vital for Ctr1 mediated high-affinity Cu-transport do not occur in COPT4 that has a non-direct role in Cu-transportation (Sancenón et al., 2004).

In addition to other processes, the transport of Cu^2+^ across the plasma membranes also involves P-type heavy metal ATPases (Williams and Mills, 2005; Takahashi et al., 2012; Yan et al., 2016). The transport of Cu into the cells may also be ascertained by the newly found cytosolic, soluble and low molecular weight heavy metal receptor proteins such as Cu chaperones (CCH), known as metallo-chaperones (O’Halloran and Culotta, 2000; Huffman and O’Halloran, 2001). Cu chaperones including COX17 (Cu chaperone for COX), CCS (Cu chaperone for SOD), and two homologs of the yeast ATX1 (antioxidant protein 1) and CCH (ATX1-like Cu chaperone) were reported to be involved in the intracellular Cu transport in *Arabidopsis* (Casareno et al., 1998; Chu et al., 2005; Puig et al., 2007). The knowledge regarding the transport of Cu into the xylem is still in its infancy. In a recent study, compared to roots developed on different metal ions the roots developed on media with 50 μM Cu exhibited a huge decline in the levels of callose (O’Lexy et al., 2018). Additionally, Cu was observed to move through plasmodesmata by influencing plasmodesmata via regulating β-1,3-glucanases.

## Copper-Induced Toxicity in Plants

A large volume of literature is available on the impact of elevated Cu on major aspects in plants including germination and growth (López-Bucio et al., 2003; Lin et al., 2005; Mench and Bes, 2009; Potters et al., 2009; Bouazizi et al., 2010; Lequeux et al., 2010; Verma et al., 2011; Feigl et al., 2013; Gang et al., 2013; Muccifora and Bellani, 2013; Adrees et al., 2015; Marques et al., 2018), photosynthesis and related variables (Chatterjee and Chatterjee, 2000; Quartacci et al., 2000; Yruela, 2005; Küpper et al., 2009; Gonzalez-Mendoza et al., 2013; Mateos-Naranjo et al., 2013; Adrees et al., 2015; Feigl et al., 2015; de Freitas et al., 2015; Emamverdian et al., 2015; Sharma et al., 2017; Ambrosini et al., 2018), phenotypic changes (Barbosa et al., 2013; Feigl et al., 2013; Sánchez-Pardo et al., 2014; Adrees et al., 2015; Nair and Chung, 2015; Ali et al., 2016; Brunetto et al., 2016; Llagostera et al., 2016; Mwamba et al., 2016; Ambrosini et al., 2018; Shiyab, 2018; Marastoni et al., 2019b; Nazir et al., 2019; Shams et al., 2019), and nutrient-use-efficiency of plants (Chatterjee and Chatterjee, 2000; Ali et al., 2002; Keutgen and Pawelzik, 2008; Ivanova et al., 2010; Feigl et al., 2013; Azeez et al., 2015; Bankaji et al., 2015; Marastoni et al., 2019a). A brief discussion on the Cu-induced changes in germination and growth, photosynthesis and related variables, phenotypic changes, and nutrient-use-efficiency of plants are presented hereunder.

### Germination and Growth

The effect of Cu was seen at different growth and developmental stages of plants from seed germination to the senescence. Contingent to Cu level and the growth stage of the test plant, excess Cu significantly affects Cu-sensitive plants. In germinating seeds, increasing Cu concentrations reduced the germination percentage of seeds in different plant species (Gang et al., 2013; Muccifora and Bellani, 2013). Similarly, germination in mungbean seeds was reported to decrease with increasing Cu concentrations (Verma et al., 2011). During an early stage of growth, elevated Cu concentrations inhibited leaf expansion but increased pigment content (Maksymiec et al., 1994; Maksymiec and Baszyński, 1996; Adrees et al., 2015). In addition to inhibition in growth and biomass, Cu toxicity in plants also includes bronzing/necrosis (Marschner, 1995; Mench and Bes, 2009; Marques et al., 2018). Increasing Cu-concentration reduces uptake of Fe, Zn, Mn, and Co (Marschner, 1995; Bouazizi et al., 2010; Feigl et al., 2013). Significant reductions in root and shoot biomass were found in *Arabidopsis* exposed to 2.5 and 5 μM Cu for 14 days (Lequeux et al., 2010). Elevated Cu can inhibit primary root growth and simultaneously stimulate lateral root formation and thereby remodel the root structure (López-Bucio et al., 2003; Potters et al., 2009; Lequeux et al., 2010). Excess Cu also causes overproduction of H_2_O_2_, which eventually weakens the cell wall-extensibility (Lin et al., 2005). Root growth is more severely affected by increased Cu than shoot growth that is obvious due to the retention of the major proportion of Cu taken up by plants.

### Photosynthesis and Related Variables

Impacts of Cu on photosynthesis are well documented. The photosynthetic apparatus is susceptible to heavy metal toxicity, which in turn directly or indirectly significantly impact photosynthetic functions. Excess Cu has been reported to decrease the level of photosynthetic pigments such as chlorophyll (Küpper et al., 2009; Ambrosini et al., 2018). Cu concentrations were reported to decrease chlorophyll content in a number of plants including spinach (Ouzounidou et al., 1998), maize (Mocquot et al., 1996), cauliflower (Chatterjee and Chatterjee, 2000), and rapeseed and Indian mustard plants (Feigl et al., 2015). Cu excess can also impair chloroplast structure and thylakoid membrane composition (de Freitas et al., 2015; Sharma et al., 2017). Ciscato et al. (1997) found that the reduction in chlorophyll biosynthesis was mainly due to Cu-exposure mediated structural damages of chloroplast particularly at the thylakoid level. Disturbed metabolic activities like loss of chloroplast integrity, and change in plastid membrane composition and inhibition of photosynthetic electron transport have also been evidenced in plants exposed to elevated Cu levels (Quartacci et al., 2000; Adrees et al., 2015). Cu was found to inhibit both PS I and PS II, where PS II was found very sensitive to elevated Cu (Yruela, 2005). In another study, excess of Cu caused a reduction in photosynthesis mainly as a consequence of the higher photoinhibition (Mateos-Naranjo et al., 2013; Adrees et al., 2015). Cu in excess may also block the photosynthetic electron transport, inhibit photophosphorylation, and decrease membrane integrity (Maksymiec et al., 1994; Emamverdian et al., 2015). Cu-excess blocked the flow of electrons from Tyr z to P680^+^ (Yruela, 2005). In *Avicennia germinans* elevated Cu-mediated 100% inhibition of net photosynthesis and reduction of chlorophyll fluorescence with damaged photosynthetic apparatus (Gonzalez-Mendoza et al., 2013). In a recent study on fibrous jute (*Corchorus capsularis*) plants, Saleem et al. (2020) reported heavy damage in the organelles of the leaves by exposure to soils having Cu-contaminated soil mixed with natural soil by 1:4 ratio. The authors also found a large number of chloroplast particles accumulated inside the cell wall and also outside the chloroplast in these plants.

### Phenotypic Changes

Surplus level of Cu restricts plant growth and development (Shams et al., 2019). The impact of Cu toxicity is primarily on root growth and phenotype, which has a paramount significance to the whole plant. In general, Cu accumulates mainly in roots rather than in shoots, although the different distribution and translocation of Cu depends on its concentration in the root-growing medium (Adrees et al., 2015). However, both shoots and roots exhibit specific symptoms of Cu-toxicity. High Cu concentrations in shoots induced pale green to white interveinal chlorosis on mature leaves, altered membrane permeability, enzyme activities and also reduced photosynthesis (Brunetto et al., 2016). In roots, excess Cu reduces root length and leads to darkening and thickening of root tips (Feigl et al., 2013). Cu stress has also been reported to decrease the area and expansions of leaves, and the size of stem in several plants (Barbosa et al., 2013; Feigl et al., 2013). In addition to reductions in shoot and root growth, elevated Cu-exposed plants exhibited phenotypic changes as toxicity symptoms, where roots showed intense dark color with increasing Cu concentration (Marastoni et al., 2019b). In several studies, elevated acquisition of Cu was culminated into chlorosis, leaf epinasty, decreased branching, thickening, and dark coloration (Nair and Chung, 2015; Ali et al., 2016) and also to the development of necrotic patches in leaf tips and margins (Llagostera et al., 2016). Altered surface root morphology, rolling of the leaf blade and reduced leaf area were also reported in plants under elevated Cu-exposure (Sánchez-Pardo et al., 2014; Nazir et al., 2019). Phytotoxic concentrations of Cu also impede the leaf proliferation, cell elasticity, and cell division and reduced the number and abundance of intercellular spaces and densely developed dark colored areas of xylem vessels (Ambrosini et al., 2018; Shiyab, 2018). Elevated Cu-accrued reduction in the leaf area resulted in reduced dry matter production (Mwamba et al., 2016). Therefore, phenotypic attributes may function as an effective bioindicator of Cu toxicity as well as for the characterization of the plants as resilient or sensitive to excess Cu.

### Nutrient-Use-Efficiency of Plants

The accumulation of ions such as Na^+^ and Cl^–^ accumulated in plant organs may compete with mineral nutrients and also disturb their uptake, translocation, and assimilation (Keutgen and Pawelzik, 2008). Higher concentrations of Cu reduced the content of N, P, and K in both shoot and root of maize; however, increased therein the concentration of Fe (Ali et al., 2002; Azeez et al., 2015). In sand culture grown cauliflower, the supply of 0.5 mM Cu for 30 days decreased Fe concentration (Chatterjee and Chatterjee, 2000). In both leaves and roots of *Brassica juncea* and *B. napus*, excess Cu impacted microelement homeostasis and decreased the concentrations of Zn, Fe, Mn, and Co (Feigl et al., 2013). Elevated Cu-mediated reduction in Zn in rapeseed has also been reported (Ivanova et al., 2010). In *Suaeda fruticosa*, Cu^2+^ increased K^+^ contents in the shoots; however, Cu^2+^ showed no effect on the level of Mg^2+^ and Na^+^ in the shoots (Bankaji et al., 2015). In a recent study on Cu-exposed oat cultivars (*Avena sativa* L. cv. Fronteira and cv. Perona), Marastoni et al. (2019a) reported a higher accumulation of Cu in the apoplasm which was argued to strongly reduce the available binding sites, leading eventually to a competitive absorption with Ca, Mn, and Zn.

### NO as a Major Plant Signaling Molecule

**FIGURE 1:**
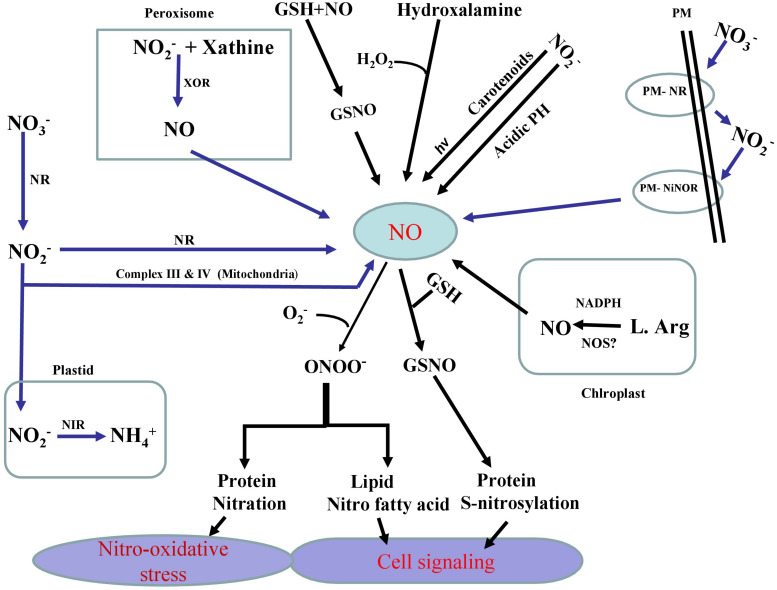
Schematic representation of the pathways (enzymatic and non-enzymatic) and their major components involved in the generation of nitric oxide (NO) in plants. Enzymatic production of NO depends on NADPH dependent oxidation of L-Arg via NO synthase (NOS)-like activity and also nitrate reductase (NR) which converts nitrate (NO_3_^–^) to nitrite (NO_2_^–^) and later (NO_2_^–^) reduction into NO via NR itself or via electron transport chain in mitochondria. Specific plasma membrane-bound nitrate and nitrite: nitric oxide reductase (PM-NR/Ni:NOR) activity utilizes NO_2_^–^ as a substrate to generate NO. Another possible route for NO formation is carried out by xanthine oxidoreductase (XOR). Non-enzymatic sources also result in the reduction of (nitrogen dioxide) NO_2_ to NO by carotenoids. In non-enzymatic pathway of NO generation that operates under sufficiently acidic medium and NO_2_^–^ gives rise NO and O_2_. NO generated in various pathways reacts with reduced GSH to produce *S*-nitrosoglutathione (GSNO), a donor and major reservoir of NO. It is also clear that NO directly modifies target proteins through reacting with reactive oxygen species (ROS) including superoxide, to generate peroxynitrite (ONOO-) which in turn causes nitrosative stress via protein tyrosine nitration. On the other, NO can also directly control cellular thiols via *S*-transnitrosylation reaction.

As a key signaling component, NO is involved in various physiological and metabolic processes in plants including their adaptation to various stresses (Asgher et al., 2017; Fancy et al., 2017; Santisree et al., 2019). In plants, NO is synthesized both by enzymatic and non-enzymatic systems (Arasimowicz and Floryszak-Wieczorek, 2007; Hasanuzzaman et al., 2018) (Figure 1). Contingent to its concentration and the site of production NO shows both positive and negative effects. Further NO was seen to affect major metabolic pathways in plants, particularly of nutrient assimilation. Our recent research on NO suggests that salt stress effects on the photosynthetic performance are mitigated effectively when NO was applied along with the split application of both N and S, and the photosynthetic activity was stimulated through increased N and S assimilation and antioxidant system conferring tolerance against salt stress (Jahan et al., 2020). Similarly. NO was also shown to reverse glucose-mediated photosynthetic inhibition in **T. aestivum L.** under salt stress (Sehar et al., 2019). We have also shown that NO can improve S-assimilation and GSH production under Cd stress and prevent inhibitory photosynthesis in mustard (Fatma et al., 2016a, b; Per et al., 2017b). Usually, NO transmits its bioactivity by targeting proteins during post-translational modifications via cysteine **S**-nitrosylation that leads to the formation of **S**-nitrosothiols (SNOs). The reaction of NO with ROS such as superoxide anions also leads to the protein tyrosine nitration and yields nitrite (ONOO-). In fact, SNOs are the key signaling molecules largely involved in response to several stresses in plant biology (Begara-Morales et al., 2019). Generated as a result of the reaction of NO with reduced GSH, **S**-nitrosoglutathione (GSNO) is the most important among SNOs. This metabolite is considered as a major reservoir of NO. As a NO-reservoir, GSNO can be transported to other cells/tissues which confer NO as a long distance mobile signaling molecule. GSNO can also be converted into oxidized glutathione (GSSG) and NH_3_ by GSNO reductase (GSNOR) (Fancy et al., 2017; Begara-Morales et al., 2018). In addition, a direct donation of the NO group of GSNO to other cellular thiols may occur via **S**-transnitrosylation reactions (Corpas et al., 2013). NO-dependent modifications in plant lipids such as nitro-fatty acids (NO_2_-FA) have shown the importance of NO in cell signaling processes (Sánchez-Calvo et al., 2013; Fazzari et al., 2014). NO_2_-FA such as linolenic acid has been reported to alleviate various abiotic stresses (Mata-Pérez et al., 2016). A NO-mediated pathway is also involved in the activation of mitogen-activated protein kinase (MAPK) signaling events (Pagnussat et al., 2004) and in the promotion of MPK6-mediated caspase-3-like activation (Ye et al., 2013).

## No in Plant Growth and Development

NO plays a vital role in regulating several processes related to plant growth and development, and qualifies the definition of plant growth regulators (Beligni and Lamattina, 2001; Xiong et al., 2009; Takahashi and Morikawa, 2014). The role of NO has been elucidated in growth and development of plants such as seed germination, senescence, root growth, leaf expansion, photo-morphogenesis, floral regulation, photosynthesis, root organogenesis, hypocotyl growth, and pathogen defense (Asgher et al., 2017; Corpas and Palma, 2018; Santisree et al., 2019), stomatal closure and the cytokinin signaling pathway (Tun et al., 2001; Desikan et al., 2004; Shi et al., 2015). Contingent to its concentration and the site of formation NO induces both positive and negative effects on plant metabolic processes. At lower concentrations, NO exhibited important positive effects in plants where it modulated germination, leaf expansion, and detoxification. On the other, several negative effects such as inhibition of photosynthesis, nitrosative stress, chlorophyll degradation were also noticed at much higher concentrations of NO (Zottini et al., 2002; Antoniou et al., 2013). In wheat seedlings, the rate of leaf expansion increased at lower concentrations of NO; however, no beneficial effect was observed at its higher concentrations (Tian and Lei, 2006). In maize, low concentration of NO promoted root growth, and its higher concentration inhibited the root growth (Lombardo et al., 2006). In a similar work, low concentration of NO (100 μM) stimulated growth of *Medicago truncatula*; whereas, a decreased growth was observed with higher concentration of NO (2.5 mM) (Filippou et al., 2013). The authors showed that 2.5 mM-mediated declines in photosynthetic rate, stomatal response, intracellular proline and putrescine accumulation and decreased *M. truncatula* growth (Filippou et al., 2013). Different dose of NO donors can differentially induce the elongation of root tips. To this end, an inhibited growth of hypocotyls in *Arabidopsis*, lettuce, and potato was observed with the treatment of 0.1 mM sodium nitroprusside (SNP), a NO-donor (Beligni and Lamattina, 2000). SNP was also reported to induce root development in *Zea mays* (Gouvea et al., 1997; Corpas and Barroso, 2015a). However, methylene blue, a NO-scavenger was reported to exhibit positive effects on the root development (Gouvea et al., 1997). Compared to GA_3_, NO was reported to exhibit its significant role in breaking the dormancy of lettuce seeds (Beligni and Lamattina, 2000). NO can also control growth and re-orientation of pollen tubes (Prado et al., 2004), induce the lateral roots mediated by the plant growth- promoting *Rhizobacterium azospirillum* (Creus et al., 2005).

The role of NO in photosynthesis has rarely been investigated. However the NO-mediated moderate improvement in photosynthetic performance of *Solanum melongena* seedlings was argued as a result of excessive quenching energy and an increase in quantum yield of PSII (Wu et al., 2013). NO with sulfur improved antioxidant defense system modulating stomatal behavior and sulfur assimilation in *Brassica juncea* (Fatma et al., 2016a). In cucumber seedlings, exogenous NO showed increased chlorophyll content, improved photosynthetic rate, transpiration rate and stomatal conductance (Fan et al., 2007). However, in *Phaseolus aureus*, NO reduced the activity of ribulose-1, 5-bisphosphate carboxylase/oxygenase (Rubisco) and increased the content of PSII oxygen-evolving complex (Lum et al., 2005). In *Kalanchoe pinnata*, NO inhibited Rubisco activity by *S*-nitrosylation in a dose-dependent manner and also slowed down photosynthetic rate (Abat et al., 2008). In *Phaseolus vulgaris* guard cells, NO showed decreased activity of the H^+^-ATPase (Ördög et al., 2013). At cellular level, NO breaks the chain reactions of oxidation and limits oxidative damage (Bakakina et al., 2014). NO can also prevent the generation of toxic hydroxyl radicals by binding with the superoxide radicals produced in the chloroplast and mitochondria during the process of electron transport chain (Arora et al., 2016). Due to its signaling nature NO was reported to upregulate the expression of certain genes to counteract oxidative damage (Jiao et al., 2016). NO also triggers the upregulation of genes such as that of chalcone synthase (CHS), glutathione-*S*-transferase (GST), alternative oxidase (AOX1a) and glutathione peroxidase (GPX) (Murgia et al., 2004). It has also been reported to deter gene expression of thylakoid ascorbate peroxidase (tAPX) controlling oxidative position of plant cell (Murgia et al., 2004).

## No-Mediated Copper-Tolerance in Plants

**FIGURE 2:**
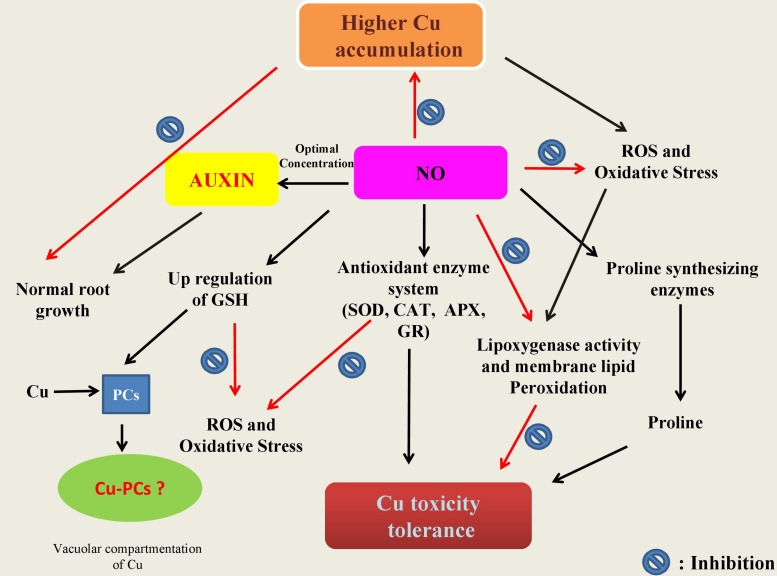
Schematic representation of the potential mechanisms underlying protective roles of nitric oxide (NO) in plants. In summary: (a) NO is involved in cellular homeostasis under Cu stress by inducing/modulating enzymes (CAT, catalase; APX, ascorbate peroxidase; GR, glutathione reductase) and non-enzyme including reduced glutathione (GSH) directly and/or indirectly involved the scavenging of reactive oxygen species (ROS); (b) NO can also be involved in the stimulation of the key enzymes of proline synthesis and thereby in modulation of the cellular proline and also in inhibition of lipoxygenase activity which in turn leads to membrane lipid peroxidation; and (c) once in cell, Cu ions make complex with phytochelatins (PCS) known to be induced by low molecular weight peptides (such as GSH) in the cytosol. The Cu-PC complexes are high molecular weight and are subsequently transported to the vacuole and thereby protect ill consequences of elevated Cu ions.

Role of NO in minimization of heavy metal stress in plants has been extensively studied (Laspina et al., 2005; Arasimowicz and Floryszak-Wieczorek, 2007; Xiong et al., 2009; Jhanji et al., 2012; Leterrier et al., 2012; Chmielowska-Bąk et al., 2014; Per et al., 2017b; Rizwan et al., 2018; Ahmad et al., 2018; Li et al., 2018) (Table 2). However, mechanisms underlying NO-mediated control of plant responses to elevated Cu-impacts are still elusive. There are two possible strategies that NO might use to mitigate heavy metal stress in plants. As the first approach, elevated Cu-exposed plants tend to upregulate their antioxidant enzymes activity or express genes involved in defense mechanism (Rizwan et al., 2018). Secondly, NO maintains the equilibrium of cellular free metal concentration either by excluding heavy metals through roots or by keeping a check on their cellular accumulation to a toxic level (Oz et al., 2015). The action mechanisms potentially involved in NO-mediated plant Cu-tolerance have been illustrated in Figure 2. The outcomes of the studies analyzing the role of NO-application in Cu-stressed plants revealed that NO reduces Cu-induced oxidative stress by increasing the activities of antioxidant enzymes; maintaining cellular redox homeostasis by elimination of ROS, and thereby promoting normal metabolic function (Cui et al., 2009; Zhang et al., 2009). The supply of sodium nitroprusside (SNP), a NO-donor to Cu-exposed *Panax ginseng* modulated the activity of H_2_O_2_-metabolizing enzymes including catalase (CAT), peroxidase, and ascorbate peroxidase (APX), and thereby increased the detoxification of H_2_O_2_ in roots (Tewari et al., 2008). Pre-treatment of *Triticum aestivum* with NO was reported to reverse the inhibitory effect of Cu stress by increasing the activity of superoxide dismutase (SOD) and CAT, and reducing the lipoxygenase activity and membrane lipid peroxidation (Hu et al., 2007). Besides inducing antioxidant system, supplied NO was also reported to promote the activity of H^+^-ATPase and H^+^-PPase in the plasma membrane or tonoplast which might play important role in tolerance to Cu stress by maintaining cytoplasmic pH (Cui et al., 2009; Zhang et al., 2009). The supplied NO-mediated escalation in the level of GSH has also been reported (Zhang et al., 2019). GSH has central role in plants in maintaining cellular redox potential (Anjum et al., 2014; Ahmad et al., 2020). Recently, Mostofa et al. (2014) reported alleviation of Cu-induced toxicity in *Oryza sativa* seedlings mainly as a result of interaction between NO and GSH. The authors revealed that the supply of SNP (200 μM) alone or in combination with GSH (200 μM) minimized Cu-impacts by reducing Cu-uptake and eased the Cu-induced oxidative damage by amending GSH production. Not only GSH content was increased but also increased the content of ascorbate (AsA), and the ratios of GSH/GSSG and AsA/DHA, which in turn strengthened antioxidant defense system improved Cu-tolerance. Moreover the contribution of different metal-chelating ligands, such as metallothioneins (MTs) and phytochelatins (PCs) are crucial players and plays pivotal role in conferring resistance to heavy metal tolerance in plants dealing with high concentrations of various metal inclusion (Cobbett and Goldsbrough, 2002; Anjum et al., 2015a; Chaudhary et al., 2018). Exogenous NO can also regulate the oxidation-reduction status of GSH-GSSG, control the GSH-PC metabolism, and also promote the vascular compartmentalization of excessive Cu in *Lemna esculentum* (Wang et al., 2018). In addition, MTs-responsive genes were induced by NO in *Solanum lycopersicum* and this NO-induced expression of MTs-related genes were reversed by NO scavenger [2-(4-carboxyphenyl)-4,4,5,5-tetramethylimidazo-line-1-oxyl-3-oxide potassium salt (cPTIO)], NOS inhibitor [*N*-nitro-l-arginine methyl ester (L-NAME)] and NR inhibitor (tungstate), which confirmed the involvement of MTs in NO-mediated tolerance to Cu toxicity (Wang et al., 2010).

**TABLE 2:** Representative studies on the copper toxicity and nitric acid role on selected parameters in plants.

**Plants**	**Copper concentration**	**Nitric oxide concentration**	**Time of nitric oxide supply**	**Parameters studied**	**Response**	**References**
*Panax ginseng*	50 μM	100 μM		Antioxidant activity (SOD, POD, APX)	+	Tewari et al., 2008
*Lycopersicon esculentum*	1.0 μM	100 μM	21 days after sowing (DAS)	H2O2, MDA	_	Wang et al., 2010
*Triticum aestivum*	5.0 mM	100 μM	3 DAS	Germination percentage, Amylase activity	+	Hu et al., 2007
*Oryza sativa*	100 μM	200 μM	12 DAS	GSH, GSSG and phytochelatins	+	Mostofa et al., 2014
*L. esculentum*	50 μM	100 μM	8 DAS	SOD, POD, CAT, APX, H^+^-ATPPase, H^+^-PPase	+	Zhang et al., 2009
Catharanthus roseus	30 mg kg-1	50 μM	30 DAS	Phenylalanine ammonia-lyase activity and total soluble phenol content,	+	Liu et al., 2016
				leaf vincristine vinblastine and total alkaloid content		
*Lolium perenne*	200 μM	100 μM		Seedlings growth	+	Dong et al., 2014
*Lepidium sativum*	50, 100, and 200 μM	50, 100 μM	20 DAS	Roots and shoots fresh weight, contents of chlorophyll *a, b*, total chlorophyll, and carotenoids	+	Raeisi et al., 2009
*O. sativa*	100 μM	200 μM	12 DAS	LOX activity, O_2_^–^, H_2_O_2_, MDA, and Proline content	_	Mostofa et al., 2014
*O. sativa*	10 mmol	100 mmol	12 DAS	NH_4_^+^ accumulation	_	Yu et al., 2005
*Nicotiana tabacum*	0.2 mM Cu	0.05 mM NO	20 DAS	Fresh weight and total chlorophyll contents,	+	Khairy et al., 2016
				Rubisco and rubisco activase activity		

*Symbols + and – indicate increase and decrease, respectively.*

Proline, a multifunctional amino acid have diverse roles in response to stress conditions, such as in stabilization of proteins, subcellular structures and membranes and protecting cellular functions by scavenging ROS potentiate plant to survive under stress (Anjum et al., 2014; Kaur and Asthir, 2015; Per et al., 2017b). The Cu-exposure accrued accumulation of proline has been found as a common response reported in several plant species (Zhang et al., 2008; Fidalgo et al., 2013; Mostofa et al., 2014). Cu-exposure caused increase in the endogenous NO production was found to modulate the cellular proline through stimulating the activity of the key proline-synthesizing enzymes such as pyrroline-5-carboxylate synthetase (Zhang et al., 2008). However, the correlation between NO content and Cu-induced proline accumulation in plants is not always apparent. For example proline content increased upon Cu-exposure but failed upon application of NO (Mostofa et al., 2014). Therefore, it indicates that the regulation of antioxidant system by NO is also dependent on the exposure conditions and the model plant. Photosynthetic functions are typically affected either directly or indirectly by elevated levels of Cu. In Cu-exposed grown *Chlorella*, NO application takes down the inhibition levels of O_2_ fixation, O_2_ evolution, and maximum quantum yield of PS II and also ominously diminished the oxidative burst (Kumar Singh et al., 2004). In another study, addition of 50, 100, 200 μM SNP protected *Lolium perenne* against Cu-toxicity as a result of increased chlorophyll content and photosynthesis, induced antioxidant enzyme activities, reduced Cu-induced oxidative damages, maintained intracellular ion equilibrium, and limited Cu translocation from roots to shoots (Dong et al., 2014). NO was found to scavenge ROS and control NH_4_^+^ accumulation in Cu-exposed *O. sativa* leaves (Yu et al., 2005). In Cu (50 μM)-exposed *Arabidopsis* seedlings, NO escalated the Cu-induced cotyledon expansion but alleviated cotyledon elongation processes (Petó et al., 2011).

In naked barley (without hull), Cu-tolerance involved nitrate reductase-mediated NO-production (Hu et al., 2015). Further NO-mediated strengthening of antioxidant defense system and the control of oxidative stress and cell death was also shown (Hu, 2016). In *Catharanthus roseus*, SNP ameliorated Cu-toxicity by decreasing the ROS-burst, promoting the contents of amino acids and the total phenolic in the roots, regulating mineral absorption and re-establishing ATPase activities (Liu et al., 2016). Yuan et al. (2013) have reported that the Cu stress in *Arabidopsis* affects root elongation by redistributing PIN1-mediated auxin (AUX). These witnessed phenotypic changes in Cu-effected roots are possibly due to AUX action, because in roots, the pattern of AUX distribution is vital for healthy root development. In addition to this Fernández-Marcos et al. (2011) showed that mutant cue1/nox1 changes NO levels, and high level of NO hampered the length of root apical meristem in *Arabidopsis*, and reduced transport of AUX and its response by altering the PIN1 levels. Therefore, the supply of optimal level of NO might be a responsible for maintaining the AUX concentration and its distribution when plants countered the heavy metal stress (Figure 2). In Arabidopsis, prolonged exposure of Cu impacted NO and AUX metabolism, and it was revealed that NO could improve Cu-induced inhibition of both root and stem growth by attenuating PIN1 induced AUX transport (Kolbert et al., 2012). In another report, Kolbert et al. (2015) established the relationship of the low Cu-sensitivity of nia1nia2noa1-2 mutant with the availability therein of low NO level and suggested that the contribution of the NR and NO associated 1-dependent pathways to NO synthesis. Thus, the above results pointed out that NO plays a vital role in response to Cu stress. However, there is still limitations in understanding the exact mechanism of NO action under Cu stress, and there is an utmost need to further investigate focusing more into molecular insights of NO action under Cu stress.

## Conclusion and Prospects

This review appraised the literature available on Cu-induced toxicity and its NO-mediated amelioration and underlying mechanisms in plants. It is clear that NO is a diffusible gaseous molecule and plays a key role in performing a number of biological functions in plants. NO acts as a signaling molecule in inducing the antioxidant system during oxidative stress. There is a remarkable progress in our understanding on the biological role of NO in plants particularly in case of salt stress. However, least information is available on the response of NO on Cu stress. Insights are required into the signaling pathways and the direct targets of NO particularly in Cu-exposed plants. Examination of the Cu-induced modulation of the NO biosynthetic pathways and its involvement in the physiological roles of NO in plants would be imperative in this regard. Some of the biosynthetic pathways of NO in plants are well known but how these pathways are interconnected and what is the mode of action in each tissue and organ are required to be elucidated.

## Author Contributions

AM and NK conceived the idea. BR, ZS, and AM prepared the first draft. NA, AM, and NK corrected and improved the manuscript. All authors approved the final version for its publication.

## Conflict of Interest

The authors declare that the research was conducted in the absence of any commercial or financial relationships that could be construed as a potential conflict of interest.
